# Low back pain and limitations of daily living in Asia: longitudinal findings in the Thai cohort study

**DOI:** 10.1186/s12891-016-1380-5

**Published:** 2017-01-19

**Authors:** Vasoontara Yiengprugsawan, Damian Hoy, Rachelle Buchbinder, Chris Bain, Sam-ang Seubsman, Adrian C. Sleigh

**Affiliations:** 1 Centre for Research on Ageing, Health and Wellbeing and Department of Global Health, Research School of Population Health, The Australian National University, Canberra, Australia; 2 Research, Evidence and Information Programme, Public Health Division, Secretariat of the Pacific Community, Noumea, New Caledonia; 3 Department of Epidemiology & Preventive Medicine, School of Public Health and Preventive Medicine, Monash University, Melbourne, Australia; 4 Monash Department of Clinical Epidemiology, Cabrini Institute, Melbourne, Australia; 5 QIMR Berghofer Medical Research Institute, Brisbane, Australia; 6 School of Human Ecology, Sukhothai Thammathirat Open University, Nonthaburi, Thailand; 7 National Centre for Epidemiology and Population Health, Research School of Population Health, The Australian National University, Canberra, Australia

**Keywords:** Low back pain, Functional limitations, Activities of daily living, Cohort study, Thailand

## Abstract

**Background:**

Low back pain (LBP) is a major cause of disability throughout the world. However, longitudinal evidence to relate low back pain and functional limitations is mostly confined to Western countries. In this study, we investigate the associations between low back pain and functional limitations in a prospective cohort of Thai adults.

**Methods:**

We analysed information from the Thai Cohort Study of adult Open University adults which included 42,785 participants in both 2009 and 2013, with the majority aged 30 to 65 years and residing nationwide. We used multivariate logistic regression to explore the longitudinal associations between LBP in 2009 and 2013 (‘never’: no LBP in 2009 or 2013; ‘reverting’: LBP in 2009 but not in 2013; ‘incident’: no LBP in 2009 but LBP in 2013; and ‘chronic’: reporting LBP at both time points) and the outcome of functional limitations relating to Activities of Daily Living (ADL) in 2013.

**Results:**

Low back pain was common with 30% of cohort members reporting low back pain in both 2009 and 2013 (‘chronic LBP’). The ‘chronic LBP’ group was more likely than the ‘never’ back pain group to report functional limitations in 2013: adjusted odds ratios 1.60 [95% Confidence Interval: 1.38–1.85] for difficulties getting dressed; 1.98 [1.71–2.30] for walking; 2.02 [1.71–2.39] for climbing stairs; and 3.80 [3.38–4.27] for bending/kneeling. Those with ‘incident LBP’ or ‘reverting LBP’ both had increased odds of functional limitations in 2013 but the odds were not generally as high.

**Conclusions:**

Our nationwide data from Thailand suggests that LBP is a frequent public health problem among economically productive age groups with adverse effects on the activities of daily living. This study adds to the limited longitudinal evidence on the substantial impact of low back pain in Southeast Asia.

**Electronic supplementary material:**

The online version of this article (doi:10.1186/s12891-016-1380-5) contains supplementary material, which is available to authorized users.

## Background

Across the adult lifespan, low back pain (LBP) is very common [1, 2]. In Western settings, evidence suggests LBP affects 40 to 60% of working adults and adversely impacts quality of life, frequently on a daily basis [3, 4]. Low back pain can lead to severe and long term impairment. The Global Burden of Disease Study listed LBP as a major cause of disability among musculoskeletal conditions and ranked LBP in the top five conditions contributing to loss of disability-adjusted life years [5, 6].

In the past decade, with its impact on productivity and activities of daily living, LBP has gained increasing attention in developing countries worldwide [2, 7]. These include, for example, a study among rural Tibetans noted LBP prevalence of 34% [8] and another study across occupation groups in Shanghai reported LBP prevalence ranging from 40% among teachers to 74% among garment workers [9]. LBP has a tendency to become chronic and a systematic review of prospective cohort studies for LBP in office workers noted previous low back pain was a key factor for subsequent pain [10]. However, longitudinal evidence relating to causes and consequences of LBP remains limited, especially in low and middle-income countries.

In this paper, we focus on LBP in a large nationwide prospective cohort of Thai adults. We investigate longitudinal associations of LBP and functional limitations of daily living. We provide an estimate of the magnitude of LBP in working adults and its consequences 4 years later, thus adding to the evidence base of low back pain globally, and in middle-income countries such as Thailand.

## Methods

This research is part of an overarching study of the health-risk transition underway in Thailand as maternal and child mortality and infectious diseases recede and chronic non-communicable diseases emerge [11]. To analyse the transition, we have developed a Thai Cohort Study enrolling 87,151 Sukhothai Thammathirat Open University distance learning adult students with a baseline 20-page comprehensive health and socio-physical-environment questionnaire in 2005. These cohort members share key sociodemographic characteristics with the general Thai population such as geographical distribution, modest median income, sex ratio, religion and ethnicity [12]. They were successfully followed up 4 and 8 years later (approximately 70% response rate in each wave; *n* = 60,569 in 2009 and *n* = 42,785 in 2013). The cohort from 2009 to 2013 is the population reported here.

### Low back pain exposure and functional limitation outcomes

In both 2009 and 2013, cohort members were asked standardised questions about LBP and if it was bad enough to limit usual activities or change daily routines for more than one day [13]. The English version of the questions and the standard diagram in 2013 are as shown in Additional file 1. It should be noted that the Thai interpretation of the standard LBP diagram initially did not cover the whole lower buttock area, but this was resolved in 2013. The age stratified crude prevalence of LBP in 2013 was slightly higher compared with 2009; among other things, this could be explained by the larger anatomical area included in the 2013 diagram.

We separately classified LBP and ‘severe’ LBP across the 4 years as longitudinal categories: ‘never’ (‘no’ in 2009 and in 2013), ‘reverting’ (‘yes’ in 2009 and ‘no’ in 2013); ‘incident’ (‘no’ in 2009 and ‘yes’ in 2013); and ‘chronic’ (‘yes’ in 2009 and in 2013).

In 2013, cohort members were asked about their functional limitations related to Activities of Daily Living (ADL) in the past 4 weeks [14]: 1) climbing stairs; 2) walking 100 metres; 3) bending, kneeling or stooping; and 4) dressing. Possible responses were: ‘not at all’; ‘a little’; ‘a lot’. The ADL questions were not connected to low back pain questions.

### Potential confounders

We collected a range of sociodemographic, behavioural and health information from cohort members at 2009 and 2013 follow-up. We also noted various other characteristics as reported in 2009 that could influence activities of daily living, including age, sex, urban-rural residence, and household monthly income (Thai Baht; 1$US ~ 30Baht). Occupation and work hours, available in the 2013 follow-up data, were used in the analyses. The average 2009–2013 values of four other covariates were used as follows: hours of standing and hours of sitting per day; physical activity (combined number of moderate or vigorous sessions per week); and body mass index (based on self-reported weight and height measurements) using recommended Asian cut-offs for overweight and obesity [15–17].

### Statistical analyses

We analyse associations between 4-year low back pain and each of the four activity of daily living outcomes as measured in 2013 (ie climbing stairs, walking 100 metres, bending/kneeling, and getting dressed). Each functional limitation model included the longitudinal 2009–2013 LBP category (never-reverting-incident-chronic) as the independent variable of interest. Multivariate logistic regression was used for analyses reporting Odds Ratios and 95% Confidence Intervals, adjusting for the potential confounders. Individuals with missing data for any given analysis were excluded (<5% for each variable), thus totals could vary slightly due to available information.

## Results

Cohort characteristics are summarised in Table 1: 45% were males; close to 80% were aged between 30 and 50 years; 55% resided in urban areas; and about 70% were managers, professionals or office workers. Table 1 also shows the prevalence of LBP and severe LBP by longitudinal categories (never, reverting, incident, chronic). For LBP, 37% were classified as ‘never’; 20% as ‘reverting’; 13% as ‘incident’; and 30% as ‘chronic’. For severe LBP, the corresponding prevalences were 95.3%, 1.7%. 2.3% and 0.7%. Notably, cohort members who had a high prevalence of ‘chronic LBP’ in both 2009 and 2013 were physical (skilled or elementary) workers (35%), with lower household income (36%), 9 h + standing daily (40%), and a body mass index of 30+ (34%).

**Table 1:** Cohort attributes by prevalence of low back pain (*N* = 42785), Thai Cohort Study

Low back pain status and cohort attributes in 2013 (%)	Longitudinal 2009–2013 low back pain dynamics^a^			
	Never ‘No’ 2009 & ‘No’ 2013	Reverting ‘Yes’ 2009 & ‘No’ 2013	Incident ‘No’ 2009 & ‘Yes’ 2013	Chronic ‘Yes’ 2009 & ‘Yes’ 2013
Low back pain	36.8 [14768]	20.0 [8055]	13.0 [5235]	30.0 [12042]
Severe low back pain restricting activities^b^	95.3 [38260]	1.7 [701]	2.3 [922]	0.7 [271]
Sex				
Male (45.1%)	38.1	19.1	13.4	29.5
Female (54.8%)	35.8	20.9	12.8	30.5
Age group, years				
< 35 (28.4%)	36.3	20.6	13.3	29.9
35–44 (45.5%)	35.8	20.5	13.2	30.4
45+ (30.2%)	38.8	19.0	12.6	29.7
Residence				
Urban (55.3%)	35.6	19.6	13.3	31.4
Rural (44.7%)	37.7	20.5	12.8	28.9
Occupation				
Professional and managers (40.3%)	38.5	20.0	13.3	27.9
Office assistant (30.5%)	36.4	20.9	12.6	30.2
Skilled worker/elementary (18.7%)	32.5	19.1	13.6	34.9
Others/not working (10.4%)	38.4	19.8	13.5	29.4
Work hours per week				
< 25 (7.2%)	34.8	19.8	13.5	31.8
25–40 (34.2%)	38.6	20.4	13.1	27.8
40+ (43.9%)	35.6	19.8	13.1	31.4
Not working (14.5%)	36.9	20.2	12.4	30.4
Household monthly income				
≤ 10,000 Baht (12.4%)	32.8	18.9	12.6	35.7
> 10,000 to 30,000 Baht (41.6%)	34.9	20.5	13.1	31.6
> 30,000 Baht (45.8%)	39.6	20.0	13.2	27.2
Hours sitting per day				
0–4 (25.4%)	36.6	20.1	12.6	30.7
5–8 (46.6%)	37.4	20.2	13.1	29.3
> 8 (27.8%)	36.1	19.9	13.4	30.6
Hours standing per day				
0–4 (70.9%)	37.4	20.0	13.3	29.3
5–8 (22.9%)	35.7	20.7	12.2	31.4
> 8 (6.1%)	33.7	19.2	13.2	33.9
Physical activity sessions per week^c^				
< 3 (50.2%)	35.7	19.9	13.5	30.9
3–6 (37.5%)	38.2	20.5	12.9	28.5
7+ (12.2%)	37.5	19.7	11.8	31.0
Body mass index (Asian cut offs)				
Underweight: BMI < 18.5 (5.7%)	37.6	21.6	12.1	28.7
Normal: BMI 18.5 to <23 (43.7%)	38.0	19.8	12.9	29.4
Overweight: BMI 23 to <25 (21.7%)	36.2	20.4	13.6	29.8
Obese I: BMI 25 to <30 (23.4%)	34.9	19.5	13.7	31.9
Obese II: BMI 30+ (5.3%)	32.4	20.7	13.0	34.0

^a^Based on status in 2009 and 2013 as follows: never, reverting, incident, and chronic

^b^‘Severe’ defined as low back pain bad enough to limit usual activities for more than one day

^c^Combined number of moderate or vigorous session (≥20 min per session)

Table 2 describes functional limitations among cohort members in 2013: approximately 6% of the cohort reported difficulty bending, 3.1% walking 100 metres, 2.2% climbing stairs, and 2.9% dressing oneself. There was a gradient of increasing functional limitation across all activities as LBP status became increasingly proximate in time (‘never’ to ‘reverting’ to ‘incident’ to ‘chronic’).

**Table 2:** Low back pain dynamics in the Thai Cohort Study (2009–2013) by functional limitations

Functional limitations	2013%	Longitudinal 2009–2013 low back pain dynamics by functional limitations, % (*n*)			
		Never ‘No’ 2009 & ‘No’ 2013	Reverting ‘Yes’ 2009 & ‘No’ 2013	Incident ‘No’ 2009 & Yes 2013	Chronic ‘Yes’ 2009 & ‘Yes’ 2013
Climbing stairs					
Never	84.5	89.9 (13225)	87.1 (6985)	81.9 (4246)	77.8 (9256)
A little	13.2	8.4 (1240)	11.0 (886)	15.6 (813)	18.9 (2257)
A lot	2.2	1.6 (236)	1.7 (140)	2.4 (126)	3.2 (383)
Walking 100 metres					
Never	81.3	87.6 (12855)	84.6 (6764)	78.1 (4050)	73.0 (8679)
A little	15.7	10.3 (1515)	13.0 (1041)	18.2 (946)	22.7 (2702)
A lot	3.1	2.1 (304)	2.3 (187)	3.5 (186)	4.1 (497)
Bending/stooping					
Never	56.7	71.5 (10519)	64.3 (5153)	45.9 (2397)	37.8 (4524)
A little	37.4	27.2 (4708)	31.6 (2537)	46.7 (2438)	51.7 (6192)
A lot	5.9	2.8 (410)	3.9 (318)	7.2 (378)	10.3 (1239)
Dressing self					
Never	82.8	88.4 (12978)	85.5 (6829)	79.1 (4105)	75.7 (8977)
A little	14.2	9.3 (1374)	11.7 (941)	17.3 (901)	20.4 (2428)
A lot	2.9	2.2 (327)	2.7 (216)	3.4 (178)	3.8 (451)

Table 3 shows the associations between 2013 functional limitations and 2009–2013 longitudinal categories of LBP (never, reverting, incident and chronic), adjusting for the potential confounders listed in Table 1. ‘Chronic’ LBP was associated with the highest odds of functional limitations: Adjusted Odds Ratios and 95% CI were 1.60 [1.38–1.85] for difficulties getting dressed; 1.98 [1.71–2.30] for walking; 2.02 [1.71–2.39] for climbing stairs; and 3.80 [3.38–4.27] for bending or kneeling. ‘Incident’ LBP had similar OR patterns but the odds were not as high - corresponding AORs for each ADL limitations were 1.49 [1.28–1.80], 1.76 [1.46–2.12], 1.53 [1.22–1.91], and 2.65 [2.29–3.06], respectively. Notable among the behavioural variables with significant associations with all ADL limitations were hours of standing (except for difficulty walking) and overweight to obese body mass index.

**Table 3:** Association between 2009 and 2013 longitudinal low back pain dynamics and 2013 functional limitations adjusting for potential covariates, Thai cohort study

Exposure variables	Outcomes - adjusted odds ratios [95% Confidence Intervals]^a^			
Low back pain category (2009 to 2013)	Climbing stairs	Walking 100 m	Bending/kneeling	Getting dressed
Never - ‘No’ in 2009 & ‘No’ in 2013	Reference	Reference	Reference	Reference
Reverting - ‘Yes’ in 2009 & ‘No’ in 2013	1.10 [0.89–1.36]	1.14 [0.95–1.38]	**1.41** [1.21–1.64]	1.18 [0.99–1.41]
Incident - ‘No in 2009 & ‘Yes’ in 2013	**1.53** [1.22–1.91]	**1.76** [1.46–2.12]	**2.65** [2.29–3.06]	**1.49** [1.28–1.80]
Chronic - ‘Yes’ in 2009 & ‘Yes’ in 2013	**2.02** [1.71–2.39]	**1.98** [1.71–2.30]	**3.80** [3.38–4.27]	**1.60** [1.38–1.85]
Covariates (2009, except^b^ and^c^)				
Sex				
Females (male - ref)	**1.19** [1.02–1.37]	1.03 [0.90–1.16]	**1.36** [1.24–1.50]	**1.39** [1.23–1.59]
Age groups (years)				
< 35	Reference			
35–44	0.91 [0.76–1.07]	0.93 [0.80–1.07]	0.98 [0.88–1.10	**0.71** [0.61–0.81]
45+	0.95 [0.78–1.15]	0.96 [0.81–1.13]	**1.35** [1.20–1.52]	**0.56** [0.47–0.66]
Residence				
Urban (rural - ref)	0.97 [0.84–1.12]	0.93 [0.82–1.05]	0.95 [0.87–1.04]	1.02 [0.90–1.15]
Occupation^b^				
Professionals and managers	Reference			
Office assistant	1.08 [0.91–1.27]	1.11 [0.96–1.29]	1.00 [0.90–1.12]	0.97 [0.84–1.13]
Skilled or elementary workers	0.79 [0.64–0.98]	1.05 [0.88–1.24]	1.13 [0.99–1.28]	0.86 [0.72–1.03]
Others/ not working	1.02 [0.73–1.41]	0.94 [0.71–1.25]	1.22 [0.98–1.51]	**0.68** [0.51–0.91]
Work hours per week^b^				
< 25	**1.54** [1.20–1.98]	**1.41** [1.14–1.76]	1.27 [1.08–1.50]	**1.55** [1.25–1.92]
25–40	Reference			
40+	1.04 [0.88–1.23]	0.96 [0.83–1.11]	1.01 [0.91–1.12]	0.94 [0.81–1.08]
Not working	**1.45** [1.08–1.95]	**1.39** [1.08–1.79]	1.14 [0.94–1.39]	**1.65** [1.28–2.11]
Household monthly income (Baht)				
≤ 10,000	1.04 [0.86–1.24]	**1.24** [1.07–1.44]	1.10 [0.98–1.24]	1.03 [0.88–1.21]
> 10,000–30,000	Reference			
> 30,000	1.01 [0.86–1.19]	0.88 [0.76–1.02]	0.90 [0.82–1.00]	0.97 [0.84–1.11]
Hours sitting per day^c^				
0–4	**1.39** [1.17–1.65]	**1.52** [1.32–1.76]	1.11 [0.98–1.24]	**1.21** [1.03–1.41]
5–8	Reference			
9+	**0.77** [0.65–0.91]	**0.83** [0.72–0.96]	0.90 [0.82–1.00]	0.82 [0.71–0.94]
Hours standing per day^c^				
0–4	1.15 [0.96–1.34]	0.95 [0.83–1.09]	1.05 [0.95–1.17]	1.17 [1.01–1.36]
5–8	Reference			
9+	**1.40** [1.07–1.84]	0.91 [0.71–1.16]	**1.33** [1.12–1.58]	**1.49** [1.18–1.89]
Physical activity (moderate or rigorous)^c^				
< 3 sessions/week	Reference			
3–6 sessions/week	0.89 [0.76–1.04]	0.91 [0.80–1.04]	**0.79** [0.72–0.87]	**0.83** [0.72–0.95]
7+ sessions/week	1.06 [0.86–1.32]	**1.32** [1.11–1.57]	0.93 [0.81–1.07]	1.05 [0.87–1.27]
Body mass index (Asian cut offs)^c^				
Underweight (<18.5)	1.06 [0.79–1.43]	1.09 [0.84–1.41]	0.89 [0.72–1.10]	**0.71** [0.51–0.97]
Normal (18.5 to <23)	Reference			
Overweight (23 to <25)	**1.25** [1.04–1.51]	**1.21** [1.03–1.42]	**1.33** [1.18–1.49]	**1.60** [1.35–1.89]
Obese I (25 to <30)	**1.42** [1.19–1.70]	**1.33** [1.13–1.55]	**1.73** [1.54–1.93]	**2.39** [2.06–2.79]
Obese II (> = 30)	**1.85** [1.43–2.40]	**1.68** [1.33–2.12]	**2.47** [2.11–2.88]	**3.03** [2.44–3.71]

^a^Bold figures were statistically significant at *p* < 0.05

^b^Covariates available in 2013

^c^Average values of covariate confounders in 2009 and 2013

Those with ‘severe’ low back pain showed similar patterns but with higher odds of functional limitations; this is not explored further due to the relative rarity of severe LBP resulting in a small sample for statistical inference.

## Discussion and conclusions

Low back pain was common in our cohort and approximately one third of participants reported LBP in both 2009 and 2013. We found an association between low back pain status from 2009 to 2013 and functional limitations for ADL in 2013, with increased limitations among those with severe low back pain. As well, ADL limitation was high among those with ‘chronic’ LBP (in both 2009 and 2013) and that limitation was greater than for those with ‘incident’ LBP, which in turn was greater than for those with ‘reverting’ LBP. The lowest ADL limitation rate was among those who had never reported LBP and we classified that as the reference rate. Such a longitudinal study of low back pain is important because it enables comparisons across time and between countries and permits causal analyses. Evidence on back pain and its effects is limited in low and middle-income countries. Similar to findings in Western economically advanced countries, our results reveal that low back pain was common and associated with clinically important limitations in activities of daily living among middle-aged and older Thai adults.

Our findings highlight the high prevalence of low back pain across all age groups in our Thai population, as reported for studies in other groups [1, 5]. Low household income was associated with both functional limitations and low back pain in our study, and this has also been reported elsewhere [2, 18]. The relationships between physical activity and presence of low back pain have also been reported previously [19–21]. We also noted an association between reduced physical activity and increased likelihood of low back pain. As well, we found an association between reduced physical activity and increasing functional limitations for ADL among Thai adults. Being obese has been reported to be associated with functional limitations [22, 23], and this was also found in our study.

We note some grounds for caution in interpreting our findings. First, our study data are drawn from a self-administered questionnaire and are subject to imperfect recall and varying individual thresholds for reporting LBP [24]. Second, while we followed the recommended international guideline for definition of low back pain for use in epidemiologic studies [13], also used by the Global Burden of Disease 2010 study [1, 5], there were slight differences in the pain diagram, with a greater area indicated in 2013. This may have resulted in higher prevalence estimates in 2013 compared with 2009. Also, our cohort was not a random population sample; even though our prevalence results may not be generalisable, the relative effects (odds ratios) should be valid. Our cohort members were open university students, all of whom had completed high school education or had equivalent experience making them better educated than average for the Thai population. On the other hand, cohort members were average working Thais in terms of their modest incomes and their geographic locations embedded in communities throughout Thailand. Lastly, we also examined the potential impact of non-response in 2013 of drop-outs (*n* = 17,784); in 2009 these were only slightly higher than the response group (2% for low back pain in 2009 and 0.5% for serious low back pain).

Another consideration is that the questions about functional limitations in activities of daily living were not linked with the question on LBP status. The ADL questions also had no direct link to LBP questions, hence our interest in hypothetical associations was not revealed. However, we cannot conclude for every individual reporting both LBP and limited ADL that there was a link. But for the population it is reasonable to see the results as showing an adverse effect of LBP on ADL given the statistical evidence that the relationship is unlikely to be due to chance and further given the clinically reasonable connection between LBP and ADL. In fact, it should be noted that our study is longitudinal and measures exposure variables at the beginning of the observation period. This design feature is an advantage with all prospective cohort studies and improves the analyses of potentially causal relationships.

Suitable lifestyle and behavioural interventions to prevent and mitigate LBP remain elusive but the high frequency of the conditions and their associated impacts warrant population health attention. Relevant LBP risk factors such as physical inactivity and obesity are complex and follow up of a prospective cohort over 10–20 years could provide insight into causal processes and mechanisms for LBP effects on population. This is particularly important for boosting knowledge in Asia for which information is limited.

LBP causes an enormous global burden, and this is generally increasing in developing countries such as Thailand. Initiatives aimed at the prevention and management of LBP, as with all musculoskeletal conditions, must be well-integrated with other non-communicable disease programs, rather than being stand alone [25]. This will avoid duplication of efforts and will help to promote a more-streamlined, cost-effective approach to overall health system strengthening.

## Additional file

Additional file 1: 2009 and 2013 Thai Cohort Study low back pain questions. (DOCX 50 kb)

## Abbreviations

ADL: Activities of Daily Living
AOR: Adjusted Odds Ratio
CI: Confidence Interval
LBP: Low Back Pain
