# Supplementary material for: Low back pain and limitations of daily living in Asia: longitudinal findings in the Thai cohort study
Source: BMC Musculoskelet Disord. 2017 Jan 19;18:19. doi: 10.1186/s12891-016-1380-5 (PMC5244554; doi:10.1186/s12891-016-1380-5)
Supplement: Additional file 1: — 2009 and 2013 Thai Cohort Study low back pain questions. (DOCX 50 kb) [file 12891_2016_1380_MOESM1_ESM.docx]

| **Supplementary file:**  **2009 and 2013 Thai Cohort Study low back pain questions**   \| **2009** \|  \| \| --- \| --- \| \| In the past 4 weeks, have you had pain in your lower back? (in the area shown on the diagram)  Yes  No  If yes, was this pain bad enough to limit your usual activities or change your daily routine for more than one day?  Yes  No \| 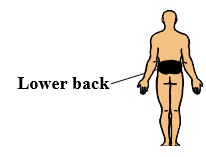 \| \| **2013** \|  \| \| In the past 4 weeks, have you had pain in your lower back? (in the area shown on the diagram)  Yes  No  If yes, was this pain bad enough to limit your usual activities or change your daily routine for more than one day?  Yes  No \|  \| |
| --- | --- | --- | --- | --- | --- | --- | --- | --- |
|  |
